# Supplementary material for: The Supplementation of Bee Bread Methanolic Extract to Egg Yolk or Soybean Lecithin Extenders Can Improve the Quality of Cryopreserved Ram Semen
Source: Cells. 2022 Oct 27;11(21):3403. doi: 10.3390/cells11213403 (PMC9658608; doi:10.3390/cells11213403)
Supplement: Supplementary file 1 [file cells-11-03403-s001.zip › cells-1978075-supplementary.pdf]

The supplementation of beebread methanolic extract to egg yolk extender or soybean lecithin extenders can improve the quality of cryopreserved ram semen

1.Total antioxidant activity; DPPH Assay

1.1. Samples preparation

1.1.1. initial screening step

Solution of sample was prepared in concentrations of 1000 and 100 μg/mL in methanol in order to identify a range within which the inhibitory concentration 50 (IC50) lies.

1.1.2. IC50 determination

Extracts that exceeded 50% inhibition in any of the initial screening step concentrations were serially diluted to provide 5 concentrations.

1.1.3. Trolox standard preparation:

a stock solution of 100μM concentration of trolox was prepared in methanol from which 7 concentration were prepared including 50, 40, 30, 20, 15, 10 and 5 μM.

1.1.4. DPPH Assay

DPPH (2,2-diphenyl-1-picryl-hydrazyl-hydrate) free radical assay was carried out according to the method of Boly, *et al.* [1]. Briefly; 100μL of freshly prepared DPPH reagent (0.1% in methanol) were added to 100μL of the sample in 96 wells plate (n=6), the reaction was incubated at room temp. for 30 min in dark. At the end of incubation time the resulting reduction in DPPH color intensity was measured at 540 nm. Data are represented as means ± SD.

1.2. Micro plate reader analysis

The results were recorded using microplate reader FluoStar Omega.

1.3. Data analysis:

Data was analyzed using *Microsoft Excel*® and the IC50 value was calculated using *Graph pad Prism 5*® by converting the concentrations to their logarithmic value and selecting non- linear inhibitor regression equation (log (inhibitor) vs. normalized response – variable slope equation)[2].

2.Total antioxidant activity; FRAP Assay

2.1. Standards and samples preparation

2.1.1. Trolox Standard for FRAP assay

Trolox stock solution of 2 mM in methanol was prepared, and 8 serial dilutions were prepared in the concentrations of 1500, 1000, 800, 600, 400, 200, 100 and 50 μM.

2.1.2. Samples preparation

samples were dissolved in 1mL DMSO and methanol was added to reach the concentration of 5mg/mL.

2.1.3. FRAP Assay

The ferric reducing ability assay was carried out according to the method of Benzie and Strain [3] with minor modifications to be carried out in microplates, briefly; A freshly prepared TPTZ reagent (300 mM Acetate Buffer (PH=3.6), 10 mM TPTZ in 40mM HCl, and 20 mMFeCl3, in a ratio of 10:1:1 v/v/v, respectively). 190 μL from the freshly prepared TPTZ reagent were mixed with 10 μL of the sample in 96 wells plate (n=3), the reaction was incubated at room temp. for 30 min in dark. At the end of incubation time the resulting blue color was measured at 593 nm. Data are represented as means ± SD.

2.2. Micro plate reader analysis

The results were recorded using microplate reader FluoStar Omega.

3. Total Phenolics and Total Flavonoids

3.1. Standards and samples preparation

3.1.1. Gallic acid standards for Total phenolics

Gallic acid stock solution of 1mg/mL in methanol was prepared, and 8 serial dilutions were prepared in the concentrations of1000, 800, 600, 400, 200, 100, 50 and 25 μg/mL.

3.1.2. Rutin standards for Total flavonoids

Rutin stock solution of 1 mg/ml in methanol was prepared, and 8 serial dilutions were prepared in the concentrations of 600, 400, 200, 100, 50, 25, 12.5 and 6,25 μg/mL.

3.1.3. Samples preparation

samples were dissolved in 1mL DMSO and the volume was completed to reach the concentration of 5mg/mL with methanol.

3.2. Micro plate reader analysis

The results were recorded using microplate reader FluoStar Omega.

3.3. Total phenolics assay (Folin-Ciocalteu)

The total phenolic content was determined using the Folin–Ciocalteu method as described by Attard [4]. Briefly, the procedure consisted of mixing 10 μL of sample/standard with 100μLof Folin-Ciocalteu reagent (Diluted 1: 10) in a 96-well microplate. Then, 80μL of 4N Na_2CO_3 was added and incubated at room temperature (25 °C) for 20min in the dark. At the end of incubation time the resulting blue complex color was measured at 630 nm. Data are represented as means ± SD.

3.4. Total flavonoids assay:

The total flavonoids content was determined using the aluminum chloride method as described by Kiranmai*, et al.* [5], with minor modifications to be carried out in microplates. Briefly, 15μL of sample/standard was placed in a 96-well microplate, then, 175μL of methanol was added followed by 30μL of 1.25 % $AlCl_3$. Finally, 30μL of 0.125 M $C_2H_3NaO_2$ was added and incubated for 5 min. At the end of incubation time the resulting yellow color was measured at 420 nm. Data are represented as means ± SD.

4. Total Soluble carbohydrates assay

4.1 Standards and samples preparation

4.1.1. Glucose standard for total soluble carbohydrates assay

Glucose stock solution of 1000 μg/mL in distilled water was prepared, and 6 serial dilutions were prepared in the concentrations of 800, 600, 400, 200, 100, and 50 μg/mL.

4.1.2. Samples preparation

100mg of sample were mixed with 10 mL distilled water and sonicated for 30 minutes at room temperature. The mixture was filtered and 1mL from the filtrate was transferred to 10mL volumetric flask and the volume was completed with distilled water obtaining the concentration of 1mg/mL.

4.1.3. Soluble carbohydrates assay

Total soluble carbohydrates assay was carried out according to the method of Gerhardt, *et al.* [6] briefly; 100μL of sample were transferred to a glass vial and mixed with 200 μL concentrated sulfuric acid solution (75% v/v). Then, 400 μL of anthrone reagent (5mg in

100μL ethanol and 2.4mL 75% v/v sulfuric acid) were added and the vial was incubated in an oven with temperature adjusted at 100°C for 15 minutes. After heating, the mixture was allowed to cool at room temperature for 5 minutes and the analysis was carried out by transferring 100 μL of the sample mixture to a 96 wells plate (n=3), where the resulting green color was measured at 578 nm. Data are represented as means ± SD.

4.2 Micro plate reader analysis

The results were recorded using microplate reader FluoStar Omega.

References

1. Boly, R.; Lamkami, T.; Lompo, M.; Dubois, J.; Guissou, I. Dpph free radical scavenging activity of two extracts from *agelanthus dodoneifolius* (loranthaceae) leaves. *International Journal of Toxicological and Pharmacological Research* **2016**, *8*, 29-34.
2. Chen, Z.; Bertin, R.; Froldi, G. Ec50 estimation of antioxidant activity in dpph assay using several statistical programs. *Food Chemistry* **2013**, *138*, 414-420.
3. Benzie, I.F.; Strain, J.J. The ferric reducing ability of plasma (frap) as a measure of "antioxidant power": The frap assay. *Analytical Biochemistry* **1996**, *239*, 70-76.
4. Attard, E. A rapid microtitre plate folin-ciocalteu method for the assessment of polyphenols. *Open Life Sciences* **2013**, *8*, 48-53.
5. Kiranmai, M.; Kumar, C.M.; Mohammed, I. Comparison of total flavanoid content of azadirachta indica root bark extracts prepared by different methods of extraction. *Research Journal of Pharmaceutical, Biological and Chemical Sciences* **2011**, *2*, 254-261.
6. Gerhardt, P.; Murray, R.; Wood, W.; Krieg, N. Methods for general and molecular bacteriology. . *American Society for Microbiology, Washington, DC* **1994**.
